# How and why community hospital clinicians document a positive screen for intimate partner violence: a cross-sectional study

**DOI:** 10.1186/1471-2296-6-48

**Published:** 2005-11-19

**Authors:** Megan R Gerber, Karen S Leiter, Richard C Hermann, David H Bor

**Affiliations:** 1 Harvard Medical School, Cambridge Health Alliance, Cambridge, MA, USA; 2 Tufts-New England Medical Center, Boston, MA, USA

## Abstract

**Background:**

This two-part study examines primary care clinicians' chart documentation and attitudes when confronted by a positive waiting room screen for intimate partner violence (IPV).

**Methods:**

Patients at community hospital-affiliated health centers completed a screening questionnaire in waiting rooms that primary care providers (PCPs) were subsequently given at the time of the visit. We first reviewed the medical records of patients who screened positive for IPV, evaluating the presence and quality of documentation. Next we administered a survey to PCPs that measured their knowledge, attitudes and practice regarding IPV.

**Results:**

Seventy-two percent of charts contained some documentation of IPV, however only 10% contained both a referral and safety plan. PCPs were more likely to refer patients (p < .05) who screened positively for mood or anxiety disorders, disclosed that they feared for their safety or were economically disadvantaged. Those that feared for their safety or endorsed mood or anxiety disorders were more likely to have notation of a safety plan in their records. When surveyed, 81.6% of clinicians strongly agreed that it is their role to inquire about IPV, but only 68% expressed confidence in their ability to manage it. In contrast, 93% expressed confidence in managing depression. Sixty-seven percent identified time constraints as a barrier to care. Predictors of PCP confidence in treating patients who have experienced IPV (p < .05) included hours of recent training and clinical experience with IPV.

**Conclusion:**

Mandatory waiting room screening for IPV does not result in high levels of referral or safety planning by PCPs. Despite the implementation of a screening process, clinicians lack confidence and time to address IPV in their patient populations suggesting that alternative methods of training and supporting PCPs need to be developed.

## Background

Intimate Partner Violence (IPV) is a major public health problem in the U.S. Nearly one-quarter of all women and 7.4% of men have been physically and/or sexually abused by an intimate partner in their adult lives [1]. Women are more likely than men to experience IPV and to be injured during an assault [1]. IPV is prevalent across geographic settings, social classes and ethnic groups [2].

Medical patients frequently have a history of IPV. In primary care practices, 5.5 to 14% of patients present with a history of recent IPV (abuse occurring in the preceding 12 months), while reported lifetime prevalence in these same settings ranges from 21 to 51% [3-5]. Women who have experienced IPV have higher rates of chronic medical conditions [6-8], and utilize more health care services [7-9].

Despite the high prevalence of IPV and its well-established adverse health impact, clinicians often do not assess their patients for IPV exposure [5,10,11]. Previous work has addressed barriers to clinician assessment for IPV. The barriers reported in the literature vary based on practice type and location, but recurring common themes include lack of time [12], fear of offending patients [10,12], fear of retaliation by the partner [11], and lack of confidence, training or inadequate resources [10,12]. Busy clinicians may simply forget to ask about IPV [10]. Despite these obstacles, most patients are willing to discuss the subject of partner violence with their medical care providers and believe that clinicians can be of assistance [13]. Abused women are even more likely than non-abused women to endorse screening [14]. Notwithstanding, universal screening for IPV remains controversial [15,16].

While prior research has examined barriers to clinician-initiated screening for IPV [10,12,17], less is known about how clinicians respond when information about a patient's IPV status is elicited for them. A better understanding of what patient or clinician characteristics drive an observed response to a positive IPV screen could inform ongoing efforts to train providers or even redirect resources in clinical settings. To address these issues, we conducted a two-part study to assess primary care provider (PCP) response to a positive screen for IPV. Using data collected in a mental health screening program at a community hospital and its affiliated health centers, we first conducted a medical record review of patients who had screened positive for IPV. Following the chart review, we surveyed PCPs to explore their attitudes and practices around intervention for patients who have experienced IPV.

## Methods

The study took place at an urban network of publicly owned community hospitals and health centers that serves an ethnically diverse population of poor and middle-income patients. In 1997, the Department of Medicine developed a program to train family practitioners, internists and primary care nurse practitioners to identify and treat patients who were victims of IPV. This program was linked to a citywide program administered by the local Department of Public Health. The training initiative included publication of a manual for PCPs and a series of three Grand Rounds presentations in 1997.

In the spring of 1998, the Department began routine screening for mental health (MH) and IPV at most of its primary care sites. A series of three IPV screening questions were appended to the MH screen which was adapted from the PRIME-MD [18]. These questions were answered in a variable time period, usually in the waiting room, prior to the encounter with the PCP. The questions were:

1. Is violence/abuse a concern in your personal relationships (past or present)?

2. Has anyone hit, pushed, slapped or threatened you in the past year?

3. Is anyone in your personal life causing you to be fearful for your safety?

Question 1 queries lifetime abuse while question 2 asks about incidents occurring in the preceding 12 months.

**Figure 1:**
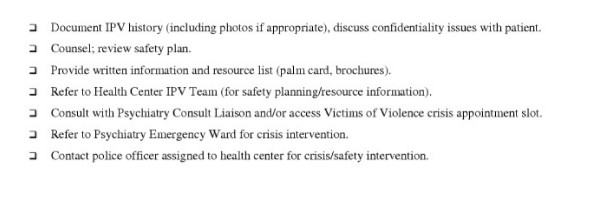
Checklist of Clinician Responses.

Clinical encounters were not delayed if a patient did not have time to complete the screen. The instrument was initially only available in English; in the second year of the study it was translated into Spanish, Portuguese and Haitian Creole. Interpreters assisted patients with completion of the questions when necessary. During the patient visit, the PCP, a nurse practitioner (NP) or physician was asked to review the mental health screen along with the responses to the IPV questions. After evaluating the patient, the provider was directed to document an assessment and plan on a separate response form, and the clinic staff faxed this form back to the Quality Management department. The response form contained a checklist intended to facilitate documentation (Figure 1). A database of screen-positive patients over the two-year period 1998–2000 was compiled.

### Chart review

In the first segment of the study, we reviewed the charts of patients who had screened positive for IPV in order to determine the nature and extent of clinician documentation following the positive IPV screen. Study standards for ideal chart documentation were derived from national consensus and state medical association guidelines that recommend detailed documentation of the circumstances of the abuse and of subsequent assessment, intervention and referral [2,19]. Thorough medical record documentation may provide important support to victims in legal settings [19]. Documentation in medical records also facilitates continuity of care between visits and among clinicians.

Four chart reviewers (two internal medicine physicians, a medical student and a dentist) abstracted data. We checked for inter-observer variability by having reviewers independently abstract sample charts: 90% agreement was demonstrated. As part of the review, we collected demographic data for both patient and provider characteristics. In addition, charts were examined for the following: any written acknowledgement of the positive IPV screen (whether on the faxed back response form or in the progress note), documentation of referral, safety planning and detailed descriptive circumstances of the abuse. Data on co-morbid psychiatric conditions were collected at the time of IPV screening and then extracted for our analysis from the same database compiled by Quality Management.

We next performed basic summary statistics analyzing patient and clinician demographic variables, psychiatric co-morbidities and chart review outcomes by response on each of the three screening questions to examine how these characteristics differed by category of IPV or concern for safety. Logistic regression analysis was then carried out to estimate two separate models examining which variables predict referral and safety planning for both lifetime and 12-month IPV.

### Clinician survey

In the second phase of the project, we designed a provider survey to characterize PCP beliefs and practices, in order to better understand the findings from the initial chart review (see Additional file 1). One of the goals of this portion of the study was to measure the impact our locally designed departmental training had on clinicians' practice, so we chose to develop our own instrument rather than use an existing one. We referenced previous work that had queried clinicians' knowledge, attitudes and practice regarding IPV in clinical practice [20-22]. Informed by prior studies, we measured our clinicians' perception of self-efficacy and sense of their role in addressing IPV [20,21]. We queried clinicians about the barriers to addressing IPV reported in previous studies [10-12,17]. Additionally drawing upon prior work, we inquired about clinicians' screening practices and their estimate of IPV prevalence among their patient panels [10,11,21]. Since we hypothesized that clinician confidence was an important factor in addressing IPV in practice, we included several items directly comparing management of IPV to other common medical and behavioral health conditions; a similar comparison has been made in other survey studies for adverse health behaviors [21]. Our survey also measured the frequency with which clinicians accessed existing hospital services when working with patients who have experienced IPV. We collected demographic data from clinicians including gender, age, and year of graduation from professional school. We also queried self-report of attendance at the original training Grand Rounds and the number of hours spent training since 1997. The survey contained scales measuring attitudes toward IPV diagnosis, perceived barriers to caring for victims, and types of intervention and resources accessed.

Between April and July of 2002, the survey was distributed and returned by interoffice mail and at a department meeting to clinicians practicing adult primary care at the same clinical sites that participated in the screening program. This sample was deemed representative of the clinicians caring for the screen-positive patients in 1998–2000. We used multivariate regression analyses to estimate predictors of PCP confidence with IPV.

The hospital institutional review board (IRB) approved the two-part study. Analyses were conducted using STATA 8.0 Statistical software (Stata Corp., College Station, TX).

## Results

### Chart review

During the two-year period 1998–2000, 4.9% (115/2341) of patients at 11 different neighborhood health centers (NHC) and primary care clinics screened positive for IPV. Of the screen-positive patients, charts of 95 (83%) were located for review. Of the 95 charts reviewed, five contained documentation of abuse that was not partner-inflicted (for example, political violence in foreign countries). These five patients were excluded from further analyses. The final number of charts eligible for review was 90.

**Figure 2:**
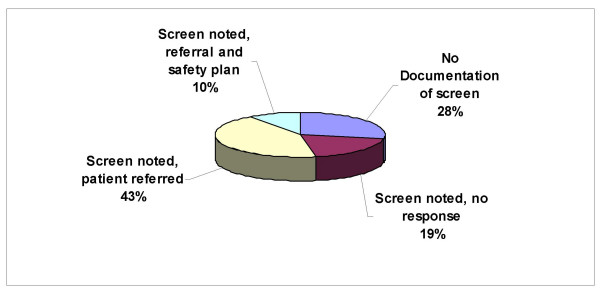
Observed Chart Documentation of IPV (N = 90).

Of the ninety IPV+ charts reviewed, 72% contained some clinician documentation of the positive screen; however, nearly one-third of the charts (28%) reviewed contained no acknowledgement of the positive IPV screen. Only nine charts (10%) contained a detailed history of the abuse along with documentation of referral and safety planning (Figure 2).

Seventy-two percent of the IPV+ patients were female and the mean age was 34 years (Table 1). Female patients comprised the majority of patients screening positive for both lifetime and 12 month IPV. The majority of IPV+ patients were white but more non-white patients endorsed a 12-month history of IPV (Table 1). Of the encounters reviewed, 64% were charted by physicians. All of the positive screens were from questionnaires completed in English. The majority of patients who screened positive for IPV were on Medicaid or Free Care (by definition these patients are of low income). More than three-quarters of the patients who answered affirmatively on the question querying fear for safety were female and of low income. The majority of patients screening positive for lifetime and 12-month IPV also screened positive for co-morbid mood and anxiety disorders. While a minority (23%) endorsed an eating disorder, more patients with 12-month IPV reported this condition.

**Table 1:** Covariates and IPV.

**Covariate**	**Total N (%)**	**Lifetime IPV** ** N = 77**	**12 month IPV** ** N = 30**	**Fear for Safety** ** N = 9**
Ethnicity White = REF	51/90 (57)	47/74 (64)	13/29 (45)	4/8 (50)
Patient Gender Female = REF	65/90 (72)	56/77 (73)	21/30 (70)	7/9 (78)
Age (mean years)	34	34	33	33
Insurance Medicaid/Free Care = REF	69/90 (77)	60/74 (81)	21/29 (72)	8/9 (89)
**Clinician Characteristics**				
Gender Female = REF	65/90 (72)	58/77 (75)	18/30 (60)	8/9 (89)
Clinician Type Physician = REF	58/90 (64)	51/77 (66)	19/29 (66)	4/9 (44)
**Psych Screen**				
Eating DO	21/90 (23)	17/74 (10)	10/29 (34)	4/9 (44)
Mood	47/90 (52)	39/74 (53)	18/29 (62)	6/9 (67)
Anxiety	59/90 (66)	50/74 (68)	21/29 (72)	8/9 (89)
**Chart Review Outcomes**				
Referral	44/90 (49)	40/76 (53)	14/30 (47)	7/9 (78)
Safety Plan	13/90 (14)	12/77 (16)	7/30 (23)	5/9 (56)

Data on the unavailable charts (n = 20) was abstracted from the hospital electronic medical record (EMR) system. The patients whose charts were unavailable for review appeared comparable to those reviewed: their mean age was 35 years and 71% were female. However, 71% of the patients whose charts were missing were described as white by the hospital EMR. Medical record number transcription error and misfiling were the most common reasons for charts to be unavailable for review. All of the sites had unavailable records, no clustering of missing charts by site was observed.

The determinants of referral and of safety planning are shown in Tables 2 and 3, respectively. We found that clinicians were more likely to refer patients (p < .05) who screened positively for mood or anxiety disorders or who had low income (Medicaid or Free Care insurance types), although the latter was not significant when adjusted for potential confounders (Table 2). Female clinicians' charts exhibited a higher proportion of documented referrals than those of their male counterparts, however this finding did not reach statistical significance. While nurse practitioners' charts had higher rates of documented referral than those of physicians, this difference also failed to achieve statistical significance.

**Table 2:** Predictors of Referral Documentation in Medical Records.

**Covariate**	**Univariate O.R. (95% C.I.)**	**Lifetime Multivariate O.R. (95% C.I.)**	**12-month Multivariate O.R. (95% C.I.)**
**Screen for IPV**			
Lifetime IPV "Is violence/abuse a concern in your personal relationships? (past or present)"	2.5 (0.70–8.8)	6.93 (1.09–43.84)	____
12-month IPV "Has anyone hit, pushed, slapped or threatened you in the past year?"	0.85 (0.35–2.0)	____	0.35 (0.94–1.30)
Fear for Safety "Is anyone in your personal life causing you to be fearful for your safety?"	4.10 (0.80–21)	2.35 (0.33–16.73)	6.31 (0.72–55.49)
**Patient Demographics**			
Age	0.980 (0.94–1.02)	0.99 (0.93–1.04)	0.99 (0.94–1.05)
Ethnicity	1.37 (0.57–2.29)	2.41 (0.76–7.66)	2.16 (0.71–6.60)
Gender Female = REF	1.35 (0.53–3.43)	0.49 (0.12–2.06)	0.54 (0.13–2.27)
Insurance Free Care/Medicaid = REF	4.70 (1.40–15.80)	2.88 (0.74–11.07)	2.83 (0.73–10.93)
**Clinician Characteristics**			
Female	2.15 (0.83–5.57)	2.53 (0.59–10.87)	2.39 (0.54–10.61)
Type Physician = REF	0.56 (.23–1.37)	0.43 (0.13–1.45)	0.51 (0.16–1.62)
Site	0.97 (.84–1.12)	0.87 (0.70–1.07)	0.89 (0.73–1.10)
**Screen for Co-morbid Psychiatric Conditions**			
Eating Disorder	.88 (.33–2.36)	________	_______
Mood	2.59 (1.08–6.20)	3.61 (1.20–10.88)	3.64 (1.24–10.7)
Anxiety	2.99 (1.15–7.73)	4.25 (1.20–15.10)	4.02 (1.18–13.73)

**Table 3:** Predictors of Safety Planning Documentation in Medical Records.

**Covariate**	**Univariate O.R. (95% C.I.)**	**Lifetime Multivariate O.R. (95% C.I.)**	**12-month Multivariate O.R. (95% C.I.)**
**Screen for IPV**			
Lifetime IPV "Is violence/abuse a concern in your personal relationships? (past or present)"	2.22 (.26–18.66)	1.14 (.05–23.72)	____
12-month IPV "Has anyone hit, pushed, slapped or threatened you in the past year?"	2.74 (.83–9.05)	____	5.66 (0.79–40.52)
Fear for Safety "Is anyone in your personal life causing you to be fearful for your safety?"	11.40 (2.54–51.31)	____	____
**Patient Demographics**			
Age	1.00 (.95–1.06)	0.98 (.92–1.05)	0.98 (.91–1.04)
Ethnicity	0.93 (.28–1.06)	1.30 (.24–7.18)	1.17 (.21–6.71)
Gender^†^	____	____	____
Insurance Free Care/Medicaid = REF	0.85 (0.21–3.49)	2.61 (.23–30.20)	2.81 (.20–38.76)
**Clinician Characteristics**			
Female	2.34 (.48–11.4)	0.25 (.01–5.90)	0.27 (.01–7.68)
Type Physician = REF	1.24 (0.35–4.40)	4.65 (.74–29.30)	5.28 (.74–37.38)
Site	1.32 (1.07–1.63)	2.18 (1.23–3.86)	2.44 (1.18–5.08)
**Screen for Co-morbid Psychiatric Conditions**			
Eating Disorder	0.93 (0.23–3.77)	0.20 (.02–2.47)	.07 (.003–1.78)
Mood	0.69 (.21–2.25)	0.08 (.01–.88)	.03 (.001–.71)
Anxiety	1.70 (.43–6.74)	15.03 (1.56–143.89)	16.45 (1.31–206.42)

^†^Only female patients' chart contained documentation of safety planning.

The results for the Safety Planning model showed a different pattern (Table 3). Patient endorsement of question 3, expressing fear for safety, was associated with safety planning documentation in charts. Only female patients' charts contained any documentation of safety planning. The site, or NHC, where screening took place was significant in both univariate and multivariate models, suggesting that safety planning practices may be site-specific. As with referral documentation, screening for the psychiatric co-morbidities of mood or anxiety appear to result in higher rates of safety planning.

### Survey

Seventy clinicians identified as eligible were surveyed. The overall response rate was 84%, 88% (40/45) for physicians and 76% (19/25) for nurse practitioners. The majority of respondents were physicians (57%). By self-report, nurse practitioners saw a mean of 10 cases annually while physicians reported 6 (p-value .057, 2-sample T-test).

The majority (81.6%) of PCPs strongly agreed that it is their role to inquire about IPV in the primary care setting. However, only 68% agreed that they were confident in their ability to diagnose and manage patients who had experienced IPV. By contrast, these clinicians reported high degrees of confidence with other medical and behavioral health conditions: 90% expressed comfort treating Chronic Obstructive Pulmonary Disease, 93% were comfortable with depression and 83% with substance abuse. A time constraint was the practice barrier endorsed most frequently in our study (67%).

The only factors significantly associated with provider confidence in IPV management were the number of hours of IPV training reported since 1997 and self report of frequency of treating patients who have experienced IPV. These two variables remained significant when adjusted for demographics and year of professional school graduation (Table 4).

**Table 4:** Determinants of provider confidence in IPV Management (Survey Data), Multivariate Linear Regression.

**Variable**	**Unadjusted β (p-value)**	**Adjusted β (p-value)**
Clinician Age	-0.24 (.124)	-0.26 (.290)
Clinician Type REF = MD	0.45 (.102)	0.22 (.521)
Years since professional school graduation	-0.02 (.241)	.004 (.871)
Provider gender REF = female	-0.21 (0.423)	-0.38 (.279)
Number hours trained since 1997 REF =< 3	-0.37 (0.029)	-0.43 (0.025)
Clinical experience with IPV	0.47 (<.001)	0.29 (0.042)

## Discussion

While prior studies in the primary care setting have focused on practice and frequency of provider-generated screening for IPV, our study evaluated an intervention that bypassed potential clinician barriers to initiating screening with use of a waiting room questionnaire. The finding that 4.9% of patients screened positive for IPV is consistent with other reports from primary care ambulatory settings [3-5]. However, the high proportion of males screening positive (31%) exceeds estimates in other studies. It is possible that some of these represent false positives. Review of chart narratives suggested that some of these male patients might actually have been batterers. Further study is needed to understand if battering behavior can be reliably detected in the primary care setting and whether detection would be efficacious.

While the majority of charts (72%) contained some documentation of the positive screen, nearly one-third of charts contained no medical record notation of the positive screen for IPV. This finding suggests that mandated waiting room screening is not sufficient to insure proper documentation and assessment by clinicians. Of the charts that did contain written acknowledgement of the positive screen, only 10% demonstrated adherence to the most complete level of documentation. While prior research has shown that medical records accurately reflect clinical reasoning [23], clinician behavior, particularly in sensitive matters, may not be fully represented by the medical record. At best, documentation is likely a proxy measure of clinicians' responses to a positive IPV screen. It is possible that appropriate care was, nevertheless, being delivered. In another study of an educational intervention in urban neighborhood health centers, documentation in the medical record did not change despite increases in the rate of screening and referral [24].

Unmeasured patient factors, such as degree of readiness to change, remain a possible explanation for the observed low rates of clinician documentation. This may be particularly true for documentation of referral and safety planning. Research applying the transtheoretical model of behavior change to abused women demonstrates that acknowledging that a relationship is abusive and accepting help often occur through a process of change [25,26]. It is possible that some of the patients identified through the screening program were not yet ready to engage in such a discussion with their clinicians or accept referrals. This phenomenon may underlie the lack of chart documentation. Possibly patient reluctance to have IPV documented may also have contributed to the low rates. Still, it is reasonable to expect that clinicians would, at a minimum, document the positive screen, even if the patient did not accept a referral or engage in a conversation about safety planning. In the earliest stage of precontemplation, research supports documenting suspicion about IPV [25].

Given the potential pitfalls of relying on medical record documentation, some investigators have recommended use of patient exit interviews as a more accurate measure of interventions for partner violence [22]. Yet for victims of IPV, medical record documentation is critical to protecting the patient's interests and safety [19] and thorough medical record documentation of IPV is recommended in national consensus guidelines [2].

When clinicians do document, our data suggests that certain patient factors – anxiety, mood, poverty and fear for safety – predict documentation by clinicians. Based on these findings, further study could determine whether questions that communicate a patient's perception of risk to clinicians result in improved documentation. Poverty may be a facilitating factor, as these patients had better insurance coverage for counseling than some privately insured patients did. Only female patients received safety planning. This finding may reflect a true subset of patients at high risk for violence and may also support the possibility that a proportion of the male patients screened falsely positive and were not at-risk victims. Gender bias on the part of clinicians is another potential explanation for this finding.

While both recent and lifetime IPV have important implications for health status, detection of recent IPV brings with it potential implications for a patient's safety. In this study, the 12-month IPV question was not predictive of referral documentation while the lifetime query was. It is unclear whether this finding relates to provider discomfort with the topic or deficiencies in training, both of which should be addressed in future work.

The provider survey results suggest that lack of confidence and experience with IPV, along with time constraints, may drive the observed variations in clinician approach to patients who have experienced IPV. Conversely, the majority of clinicians expressed comfort in their ability to manage depression and substance abuse. This difference is notable since these conditions, like IPV, have been considered sensitive and of a private nature in the past and might still carry a stigma for some patients.

### Limitations

Our study has a number of limitations. The screening questions were not validated and generated false positive screens for IPV. The questions were narrow in scope and did not address the dimensions of emotional or sexual abuse, possibly failing to identify an important segment of the target population, and likely resulting in an underestimate of the true prevalence of IPV in our population. This may also impact the external validity of the study. Our sample was a convenience sample and we do not know the total number of patients who received the questionnaires in waiting rooms; the only screens counted were those returned by the clinical sites. Distributing the screening questions in the waiting room may have resulted in false negative responses, as some of the patients may have been accompanied by a battering partner or may have had concerns for confidentiality. The lack of positive screens on any instrument translated into another language raises concerns about literacy and cultural barriers to written disclosure of sensitive information. Data on gender preference was often not available in charts rendering us unable to determine whether a percentage of the males screening positive were men who have sex with men. In addition, PCP survey responses may have been biased to comply with the department's strongly stated position on the importance of IPV recognition and intervention. These responses may not be generalizeable to other departments that lack such a clear mandate. The urban setting of the study may also limit the generalizeability of our results to institutions in non-urban areas. Finally, our sample size was small, limiting the ability to detect statistically significant relationships among the variables and producing wide confidence intervals for some of the estimates.

## Conclusion

When prompted by a positive IPV screen, clinicians frequently address this issue; however their chart documentation (and possibly their approach) is inadequate. They express less confidence in their ability to address IPV than other medical conditions that carry a similar social stigma. Why are clinicians so much more confident in managing other potentially stigmatizing conditions, such as substance abuse? One possible explanation is a shift in professional mores: conditions such as this one have evolved from being considered private, personal afflictions to diseases with a clear health impact. Only after a shift in the way these conditions were regarded did strategies for effective training and proven intervention evolve. It is only in the last decade that IPV has been recognized as a significant medical concern [27]. Alternative approaches are necessary to assist clinicians in adequately addressing IPV in office practice; as the results of this study demonstrate, waiting room screening alone is not sufficient. Potential areas for future investigation include the development and evaluation of experiential educational methods for clinicians and identification of the optimum frequency for retraining PCPs in proper documentation, referral and safety planning. Electronic medical record prompts may also aid busy clinicians and provide seamless, legible documentation of IPV. It is incumbent upon primary care clinicians to improve the identification and quality of care for the large number of patients they routinely see who experience partner violence.

## Competing interests

The author(s) declare that they have no competing interests.

## Authors' contributions

MRG designed the study, oversaw data collection, performed the statistical analyses and drafted the manuscript. KSL participated in the design of the study and helped draft the manuscript. RCH contributed to data interpretation and manuscript revisions. DHB conceived of the study, contributed to data interpretation and helped revise the manuscript. All authors have read and approved the final manuscript.

## Pre-publication history

The pre-publication history for this paper can be accessed here:



## Supplementary Material

Additional File 1 Clinician survey uploaded as Additional File 1. Click here for file
