# Supplementary material for: How and why community hospital clinicians document a positive screen for intimate partner violence: a cross-sectional study
Source: BMC Fam Pract. 2005 Nov 19;6:48. doi: 10.1186/1471-2296-6-48 (PMC1318461; doi:10.1186/1471-2296-6-48)
Supplement: Additional File 1 — Clinician survey uploaded as Additional File 1. [file 1471-2296-6-48-S1.doc]

# DOMESTIC VIOLENCE PROVIDER SURVEY

# Section A: Demographic Information

|  |  |
| --- | --- |
| 1. Age | 25-35  36-45  46-55  56-65  > 65 |
| 2. Gender | Female  Male |
| 3. Ethnicity | White  Black  Asian  Latino  Other |
| 4. Provider Type | MD  NP  PA  Other |
| 5. What year did you graduate from your professional school? | |
| 6. What year did you join Cambridge Health Alliance (CHA)? | |
| 7. Please estimate the number of cases of domestic violence you saw in the past year (Jan ’01 – Jan ’02) | |
|  | |

# Section B: Provider Training

| 1. I had training on domestic violence prior to screen implementation (March 1998). | Yes | No  Please skip to B4 | Do Not Remember  Please skip to B4 | |
| --- | --- | --- | --- | --- |
| 2. Type of training received?  Mark all that apply. | Grand Rounds | CME | Professional School | Other |
| 3. Estimated total number of hours  spent on domestic violence training prior to March 1998 | < 3 Hrs. | 3-6 Hrs. | > 6 Hrs. |  |
| 4. I attended the domestic violence Grand Rounds in 1997 at CHA. | Yes | No  Please skip to B6 | Do Not Remember  Please skip to B6 | |
| 5. Number of domestic violence  Grand Rounds Attended at CHA in 1997 | 1 | 2 | 3 | Do Not Remember |
| 6. I have received training on domestic violence **since** the 1997 Grand Rounds. | Yes | No  Please Skip to C1 | Do Not Remember  Please Skip to C1 | |
| 7. Type of Training | Grand Rounds | CME | Professional School | Other |
| 8.Estimated number of hours spent on training since 1997 | < 3 Hrs. | 3-6 Hrs. | > 6 Hrs. |  |

# Section C: Attitudes

Please rate your agreement or disagreement with the following statements according to the scale provided.

|  | **Agree Strongly** | **Agree** | **Neutral** | **Disagree** | **Disagree Strongly** |
| --- | --- | --- | --- | --- | --- |
| 1. Domestic violence is an important healthcare issue. |  |  |  |  |  |
| 2. The primary care clinic is not the appropriate place to ask about domestic violence. |  |  |  |  |  |
| 3. Domestic violence is a public health problem similar to… |  | | | | |
| a. Diabetes |  |  |  |  |  |
| b.Tobacco Abuse |  |  |  |  |  |
| 4. I believe that it is my role to inquire about domestic violence in the primary care setting. |  |  |  |  |  |
| 5. I believe that effective intervention for domestic violence is possible in the primary care setting. |  |  |  |  |  |

**Section D: Experience**

Please rate your agreement or disagreement with the following statements according to the scale provided.

|  | **Agree Strongly** | **Agree** | **Neutral** | **Disagree** | **Disagree Strongly** |
| --- | --- | --- | --- | --- | --- |
| 1. In my practice I frequently diagnose and manage patients with… |  | | | | |
| COPD |  |  |  |  |  |
| Menopausal Symptoms |  |  |  |  |  |
| Depression |  |  |  |  |  |
| Substance Abuse Disorders |  |  |  |  |  |
| Domestic Violence |  |  |  |  |  |
| 2. I am confident in my ability to diagnose and manage patients with…. |  | | | | |
| COPD |  |  |  |  |  |
| Menopausal Symptoms |  |  |  |  |  |
| Depression |  |  |  |  |  |
| Substance Abuse Disorders |  |  |  |  |  |
| Domestic Violence |  |  |  |  |  |

# Section E: Barriers

Please rate your agreement or disagreement with the following statements according to the scale provided.

|  | **Agree Strongly** | **Agree** | **Neutral** | **Disagree** | **Disagree Strongly** |
| --- | --- | --- | --- | --- | --- |
| 1. Lack of time is a barrier to counseling patients that screen positive for domestic violence in my clinic. |  |  |  |  |  |
| 2. My training is inadequate to treat victims once they are identified. |  |  |  |  |  |
| 3. I have adequate professional staff in my clinic to help me manage domestic violence. |  |  |  |  |  |
| 4. I worry that I will offend my patients by asking about domestic violence. |  |  |  |  |  |
| 5. I am concerned about my own personal safety when treating victims of domestic violence. |  |  |  |  |  |
| 6. Discussing domestic violence with my patient may escalate his/her risk of abuse. |  |  |  |  |  |
| 7. Insurance reimbursement rates are a barrier to treating domestic violence patients |  |  |  |  |  |
| 8. The legal ramifications of addressing domestic violence is a barrier to treating victims. |  |  |  |  |  |
| 9. My personal experiences make it difficult for me to ask patients about domestic violence. |  |  |  |  |  |

# Section F: Screening Practices

Please answer the following questions/statements concerning the use of the “pink” Mental Health screening forms.

| 1. Has your practice site ever used the Mental Health screening form? | Yes | | No | |
| --- | --- | --- | --- | --- |
| 2. At what kinds of visits do you see a completed screening form? **Check all that apply.** | New Patient Visits | Physicals | | Other |
| 3. How much time does it take for you to document domestic violence? | < 1min. | 1-5 minutes | | > 5 minutes |
| Do you feel it takes too long to document? | Yes | | No | |

# Section G: Screening and Management Form

Please rate your agreement or disagreement with the following questions concerning the CHA “Pink” Screening Form for domestic violence.

|  | **Agree Strongly** | **Agree** | **Neutral** | **Disagree** | **Disagree Strongly** |
| --- | --- | --- | --- | --- | --- |
| 1. After implementing the domestic violence management form…. |  | | | | |
| (a) The number of patients I have counseled for domestic violence has increased |  |  |  |  |  |
| (b) My ability to manage domestic violence cases has improved due to the domestic violence management form |  |  |  |  |  |
| (c) My ability to refer patients for treatment has improved due to the domestic violence management form |  |  |  |  |  |
| 2. Preprinted aids such as action checklists and body maps make documentation easier. |  |  |  |  |  |
| 3. I believe the **mental health screening** form is able to pick up true cases of domestic violence most of the time (~75% true positive cases) |  |  |  |  |  |
| 4. I believe all patients who screen negative on the **mental health screening** form are truly free from domestic violence most of the time (~75% true negative cases) |  |  |  |  |  |
| 5. I routinely ask my patients about domestic violence independent of the **mental health screening form**. |  |  |  |  |  |

# Section H: Services Provided by CHA

Please rate the frequency in which you are able to obtain referrals to the following services.

|  | **Agree Strongly** | **Agree** | **Neutral** | **Disagree** | **Disagree Strongly** |
| --- | --- | --- | --- | --- | --- |
| 1. I refer patients who screen positive for domestic violence to…. |  | | | | |
| a. The clinic social worker |  |  |  |  |  |
| b. Victims of Violence |  |  |  |  |  |
| c. On-Site Psychiatric Liaison Service |  |  |  |  |  |
| d. Other |  |  |  |  |  |
| 2. It is easy for me to refer patients who screen positive for domestic violence to…. |  |  |  |  |  |
| a. The clinic social worker |  |  |  |  |  |
| b. Victims of Violence |  |  |  |  |  |
| c. On-Site Psychiatric Liaison Service |  |  |  |  |  |
| d. Other |  |  |  |  |  |
| 3. Prompt evaluation of patients who screen positive for domestic violence is possible at CHA |  |  |  |  |  |
| 4. If a patient screens positive for domestic violence and currently sees a mental health provider, I notify that provider of a positive screen |  |  |  |  |  |

# Section I: Comments

Please provide any additional comments concerning the current domestic violence protocol at CHA in the space provided below and on the next page.

|  |
| --- |
|  |
|  |
|  |
|  |
|  |
|  |
|  |
|  |
|  |
|  |
|  |
|  |
|  |

**END OF SURVEY**

**Thank you for your time and input to our study.**
